# A Novel Protein-Protein Interaction in the RES (REtention and Splicing) Complex*

**DOI:** 10.1074/jbc.M114.592311

**Published:** 2014-08-26

**Authors:** Konstantinos Tripsianes, Anders Friberg, Charlotte Barrandon, Mark Brooks, Herman van Tilbeurgh, Bertrand Seraphin, Michael Sattler

**Affiliations:** From the ‡ Central European Institute of Technology (CEITEC), Masaryk University, Kamenice 5, 62500 Brno, Czech Republic,; the § Institute of Structural Biology, Helmholtz Zentrum München, Ingolstädter Landstr. 1, 85764 Neuherberg, Germany,; the ¶ Center for Integrated Protein Science Munich and Chair of Biomolecular NMR, TU München, Lichtenbergstr. 4, 85747 Garching, Germany,; the ‖ Centre de Génétique Moléculaire, CNRS, Avenue de la Terrasse, 91198 Gif sur Yvette, France,; the ** University Paris-Sud, Institut de Biochimie et de Biophysique Moléculaire et Cellulaire, UMR8619, F-91405 Orsay, France, and; the ‡‡ Equipe Labellisée La Ligue, Institut de Génétique et de Biologie Moléculaire et Cellulaire (IGBMC), Centre National de Recherche Scientifique (CNRS) UMR 7104, Institut National de Santé et de Recherche Médicale (INSERM) U964, Université de Strasbourg, 67404 Illkirch, France

**Keywords:** Gene Regulation, Protein Complex, Protein Structure, Protein-protein Interaction, RNA-binding Protein, Spliceosome, U2AF Homology Motif

## Abstract

The retention and splicing (RES) complex is a conserved spliceosome-associated module that was shown to enhance splicing of a subset of transcripts and promote the nuclear retention of unspliced pre-mRNAs in yeast. The heterotrimeric RES complex is organized around the Snu17p protein that binds to both the Bud13p and Pml1p subunits. Snu17p exhibits an RRM domain that resembles a U2AF homology motif (UHM) and Bud13p harbors a Trp residue reminiscent of an UHM-ligand motif (ULM). It has therefore been proposed that the interaction between Snu17p and Bud13p resembles canonical UHM-ULM complexes. Here, we have used biochemical and NMR structural analysis to characterize the structure of the yeast Snu17p-Bud13p complex. Unlike known UHMs that sequester the Trp residue of the ULM ligand in a hydrophobic pocket, Snu17p and Bud13p utilize a large interaction surface formed around the two helices of the Snu17p domain. In total 18 residues of the Bud13p ligand wrap around the Snu17p helical surface in an U-turn-like arrangement. The invariant Trp^232^ in Bud13p is located in the center of the turn, and contacts surface residues of Snu17p. The structural data are supported by mutational analysis and indicate that Snu17p provides an extended binding surface with Bud13p that is notably distinct from canonical UHM-ULM interactions. Our data highlight structural diversity in RRM-protein interactions, analogous to the one seen for nucleic acid interactions.

## Teaser

**Background:** The heterotrimeric retention and splicing (RES) complex is conserved from yeast to man.

**Results:** Structural and biochemical analyses show an extended binding interface between the Snu17p RNA recognition motif (RRM) and Bud13p of the RES complex.

**Conclusion:** The Snu17p RRM-Bud13p interaction represents a novel RRM-protein interface.

**Significance:** The Snu17p-Bud13p complex may provide a platform for additional protein and RNA interactions in the RES complex.

## Introduction

RNA splicing is one of the fundamental processes in constitutive and regulated gene expression in eukaryotes. During splicing, introns present in primary transcripts (pre-mRNAs) are removed and exons are ligated to produce translationally competent mRNAs. The reaction is mediated by the spliceosome, a highly dynamic machinery of five small nuclear ribonucleoprotein particles (snRNPs)^6^ and a large number of non-snRNP interacting proteins (1). In the course of the splicing reaction snRNPs assemble stepwise and undergo substantial conformational rearrangements that allows processing of a wide variety of transcripts. In addition, non-snRNP proteins associate and exchange at different stages of the splicing process, allowing for further splicing regulation or coupling of splicing to other cellular processes (2).

A non-snRNP complex has been recently identified in *Saccharomyces cerevisiae* that enhances pre-mRNA splicing and prevents leakage of unspliced pre-mRNAs from the nucleus (3). The yeast pre-mRNA retention and splicing complex, called the RES complex, consists of Snu17p, Bud13p, and Pml1p proteins. Snu17p acts as a central platform, which independently binds Bud13p and Pml1p (4–6). The RES subunits and their human orthologs have been shown to associate transiently with the spliceosome prior to the first catalytic step that leads to intron excision (7, 8). Yeast strains carrying deletions of *snu17*, *bud13*, or *pml1* are viable but exhibit slow growth, a phenotype exacerbated at high temperatures. *In vitro* and *in vivo* splicing assays have demonstrated that the RES complex enhances splicing, especially of those introns that contain poor consensus sequence at the 5′ splice site (3, 9), including alternatively spliced genes (10). Inactivation of RES subunits also induces leakage of pre-mRNAs from the nucleus to the cytoplasm, indicating that the RES complex has an important role in nuclear retention of unspliced transcripts (3). Finally, the RES complex was recently shown to facilitate the splicing of introns with short 5′ splice site-branch point distances (11) and to interact genetically with the enzyme mediating trimethylation of the U snRNAs (12).

**FIGURE 1.**
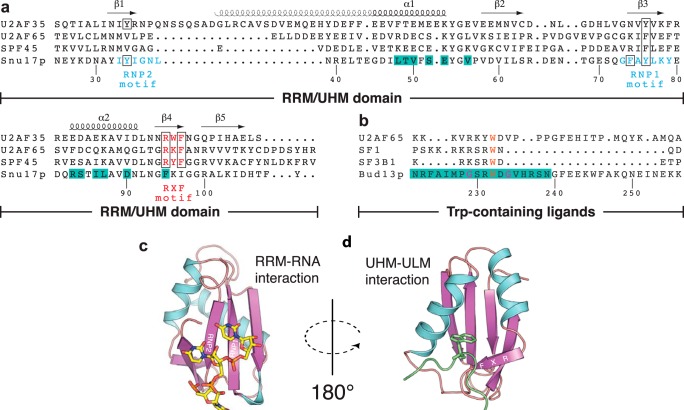
**Sequence and structural features of RRM and UHM domains.** *a*, structure-based sequence alignment of UHM/RRM domains. Secondary elements are indicated on the *top* and residue numbers of Snu17p are given at the *bottom*. Residues in the RNP motifs of Snu17p and Arg-*X*-Phe motifs of UHM domains are colored *cyan* and *red*, respectively. Conserved residues in the motifs are highlighted by *black boxes. b,* sequence alignment of ULM peptide sequences known to bind UHM domains with the Bud13p ligand (residue numbers of Bud13p indicated at the bottom). The invariant tryptophan of ULM ligands and the two glycine residues that are unique to Bud13p are colored *orange* and *magenta*, respectively. Residues that form the binding interface between Snu17p and Bud13p are highlighted with a *lime green background* in *a* and *b*, respectively. *c* and *d,* structures of a canonical RRM domain (PDB code 2G4B) in complex with RNA (*c*) and a representative UHM domain (PDB 2PEH) bound to a ULM peptide (*d*). RNP motifs and the UHM-specific RXF sequence are annotated in β strands.

Of the three RES subunits, Snu17p contains an RNA recognition motif (RRM) domain (Fig. 1). The RRM motif is the most abundant RNA-binding domain in higher vertebrates and found in many spliceosomal proteins. RRMs usually exhibit a compact-fold comprised of four- or five-stranded β-sheet that represents the RNA binding surface and two α helices packed against the β-sheet. Sequence conservation between various RRMs is low except for three aromatic side chains belonging to the two signature sequences RNP1 and RNP2, which are responsible for RNA binding (Fig. 1, *a* and *c*) (13, 14). The UHM (U2AF homology motif) domain is a variant of the RRM domain and has been shown to mediate protein-protein interactions via the α-helical face of the RRM-fold (15). UHM domains often lack aromatic residues in the RNP signature sequences and thus do not or only poorly bind to RNA. An Arg-*X*-Phe motif in UHM domain is involved in the recognition of a conserved tryptophan residue in the UHM ligand motifs (ULMs). Structural studies have demonstrated a common mode of ligand recognition by different UHM domains (15–19). The invariant Trp residue in the ULM (Fig. 1*b*) inserts into a tight hydrophobic pocket formed by the two helices and the Arg-*X*-Phe motif of the UHM domain (Fig. 1*d*), whereas positively charged residues preceding the conserved Trp in the ULM motif interact with negatively charged residues on the UHM domain.

Previous biochemical studies have indicated that the RRM domain of Snu17p is sufficient for binding Bud13p. Although some unconventional features of the Snu17p domain were noticed, such as the absence of the Arg-*X*-Phe motif (Fig. 1*a*), it has been proposed that the Snu17p-Bud13p interaction follows the structural characteristics of UHM-ULM interactions (4, 6, 20). To better understand how the RES complex is organized to perform its biological functions we have determined the binary complex structure between Snu17p and Bud13p. Isothermal titration calorimetry (ITC) together with NMR binding experiments allowed the identification of the minimal length of the Bud13p ligand. The NMR-derived solution structure shows that Snu17p and Bud13p do not interact through an UHM-ULM type of recognition. Rather, the interaction involves 18 residues of Bud13p that contact the helical surface of the Snu17p RRM domain. Notably, the invariant Trp of Bud13p is not buried in a hydrophobic cavity, distinct from canonical UHM-ULM interactions. The structural data are supported by mutational analysis of the interacting partners testing for their ability to form the RES complex when co-expressed in *Escherichia coli* or to restore the growth phenotype of Δ*snu17* or Δ*bud13* strains.

## EXPERIMENTAL PROCEDURES

### 

#### 

##### Cloning, Protein Expression, and Purification

Snu17p proteins (residues 1–113 or 25–113) and Bud13p proteins (residues 201–266 or 222–256) were cloned into a modified pET-24d vector using standard protocols. The fusion proteins expressed from these vectors comprise a His_6_ tag followed by a GST fusion domain and a tobacco etch virus proteolytic cleavage site. Proteins were expressed in *Escherichia coli* BL21(DE3) pLysS (Novagen) using kanamycin for selection. A 10-ml Luria broth (LB) pre-culture was inoculated with a single colony from a transformation plate. The pre-culture was used to start larger 1-liter cultures, containing LB or M9 minimal medium for labeling with ^15^N or ^15^N/^13^C. Upon reaching optical density of 0.6 cultures were induced with 0.5 mm isopropyl β-d-1-thiogalactopyranoside for 4 h at 37 °C.

Recombinant proteins were purified by sonicating the harvested cell pellet in 25 ml of lysis buffer (20 mm Tris, pH 7.5, 300 mm NaCl, 10 mm imidazole, 1 mm DTT, and 0.02% NaN_3_), also including protease inhibitors, RNase, lysozyme, and 0.2% IGEPAL. After high speed centrifugation (20,000 × *g*, 30 min) and filtering, each supernatant was applied to Ni-NTA-agarose resin (Qiagen). Several rounds of washing were performed with: lysis buffer including 0.2% IGEPAL, lysis buffer, lysis buffer with high salt concentration (1 m NaCl), and lysis buffer with high imidazole concentration (30 mm imidazole). Finally, each protein was eluted by applying 10 ml of a buffer containing 20 mm Tris, pH 7.5, 300 mm NaCl, 330 mm imidazole, 1 mm DTT, and 0.02% NaN_3_. Tobacco etch virus protease was added to each sample and incubated overnight at 4 °C. To remove the cleaved GST tag each sample was passed over a second Ni-NTA column. A second purification step included size exclusion chromatography (HiLoad, Superdex 75 16/60, GE Healthcare). In this step, proteins were buffer exchanged into the NMR buffer (20 mm sodium phosphate, pH 6.3, 25 mm NaCl, and 2 mm fresh DTT or Tris(2-carboxyethyl)phosphine, 0.02% NaN_3_). Stoichiometric hybrid-labeled samples were prepared by mixing excess Bud13p with Snu17p and performing another step of size exclusion chromatography (HiLoad, Superdex 75 16/60, GE Healthcare). Two shorter peptides of Bud13p (residues 221–242 or residues 225–238) were purchased from Peptide Specialty Laboratories (Heidelberg, Germany), and extensively dialyzed before binding experiments.

An operon system encoding His_6_-Snu17p, Bud13p, and Pml1p was described earlier (4). Snu17p and Bud13p variants were introduced into this vector by standard mutagenesis and cloning procedures. Protein expression, complex mini-purifications, and denaturing gel analyses of complex composition were performed as previously described (4).

##### NMR Spectroscopy and Structure Calculation

NMR spectra were recorded at 298 K on an AVIII800 Bruker NMR spectrometer equipped with a cryogenic triple resonance gradient probe. Spectra were processed using NMRPipe (21). Protein backbone and side chain assignments were obtained from standard triple resonance experiments (22). NOE distance restraints were obtained from ^15^N- and ^13^C-edited three-dimensional NOESY spectra (mixing time 70 ms) where only Snu17p or Bud13p proteins were labeled. Intermolecular NOE restraints were derived from three-dimensional ^15^N,^13^C-filtered, ^13^C-edited NOESY spectra (mixing time 70 ms) (22, 23). Stereospecific assignments of pro-chiral methyl groups of valine and leucine were obtained using 10% ^13^C-labeled protein samples (24).

Structure calculations with torsion angle dynamics were performed by using the program CYANA 3.0 (25). For every assigned peak, the distances between the protons (for a range between 2.4 and 6 Å) were calibrated using the calibration algorithm of CYANA. The set of NOE distance restraints together with ϕ and ψ backbone dihedral angle restraints derived from TALOS+ (26) based on the chemical shifts were used for water refinement (27) using CNS (28). The structure was validated using iCing. Molecular images were generated with PyMol (Schrödinger).

##### Isothermal Titration Calorimetry

Calorimetric measurements were performed with a VP-ITC (MicroCal Inc.) instrument at 25 °C using samples in NMR buffer. The concentration of Snu17p (residues 1–113) in the cell (1440 μl) varied between 30 and 70 μm, whereas the syringe (300 μl) contained Bud13p ligands at concentrations ranging from 0.6 to 1.1 mm. Normally 50 injections were done over a period of 3–4 h. Bud13p ligands were also injected into buffer and the heat of dilution control was subtracted from the titration. Data fitting was done in Origin v7 using one- or two-step binding models supplied by the manufacturer. The dissociation constant values are given with the fitting error.

##### In Vivo Analyses

The wild type *snu17* and *bud13* genes were cloned in the pRS425 plasmid backbone. The TAP tag coding sequence (29) was introduced to produce C-terminal fusions, allowing the monitoring of protein expression levels. *snu17* and *bud13* variants were introduced into the resulting vectors by standard mutagenesis and cloning procedures. These plasmids were transformed, respectively, into Δ*snu17* or Δ*bud13* strains (3, 4). Transformants were selected and complementation was assayed by spotting serial dilutions of the strain on selective plates and incubating at the indicated temperatures. Expression of wild type and mutant proteins was monitored by extracting total proteins and monitoring the levels of the TAP-tagged fusions by Western blotting.

## RESULTS

### 

#### 

##### RES Complex Assembly and Function

Previous biochemical analyses have shown that Snu17p binds Bud13p directly (4, 6). Based on a mutational analysis of the invariant Bud13p Trp^232^ residue (6), the interaction of Bud13p with the Snu17p RRM has been proposed to resemble a typical UHM-ULM interaction. Another study, however, showed that the W232A mutation did not abolish its interaction with Snu17p (4), in contrast to what has been reported so far for UHM-ULM interactions (18). Moreover, it had been noticed that Snu17p lacks the UHM characteristic Arg-*X*-Phe motif and also contains the RRM-typical consensus sequence motifs (RNP1 Phe^74^, Tyr^76^, and RNP2 Tyr^34^) that mediate RNA interactions in canonical RRM domains (Fig. 1, *a* and *c*).

**FIGURE 2.**
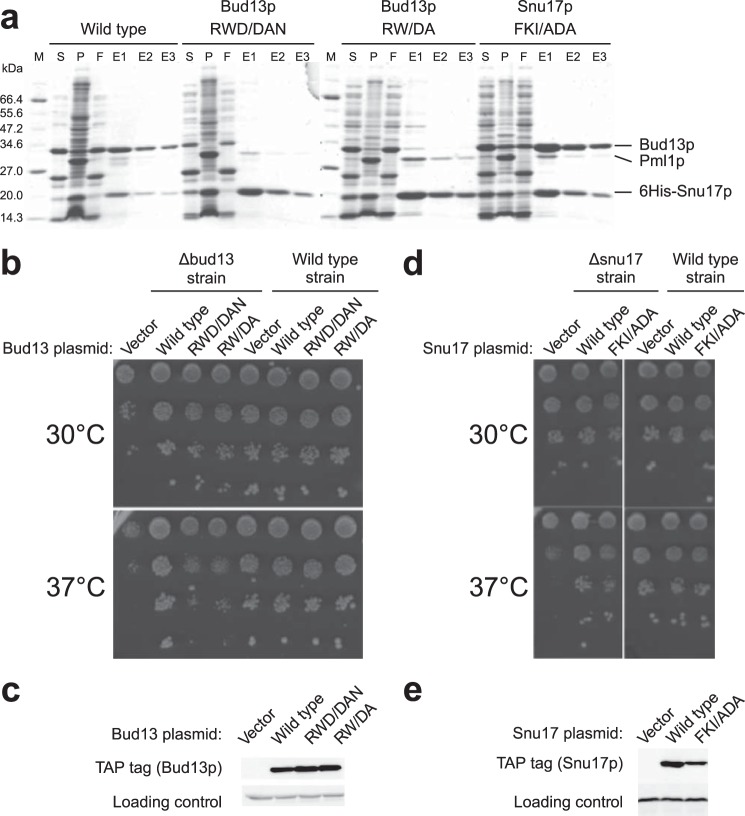
**Mutational analyses of the Bud13p-Snu17p interaction.** *a,* interactions between Snu17p, Bud13p, and Pml1p were assayed by one-step Ni-NTA purification of wild type or mutant proteins co-expressed in *E. coli*. Supernatant (*S*) and pellet (*P*) fractions from bacterial lysates as well as flow-through (*F*) and elution (E1, E2, and E3) fractions from the Ni-NTA purifications are shown after Coomassie staining of the gels. *M* indicates the molecular mass marker with size indicated on the *left* in kDa. Note that Pml1p is less well stained than Snu17p and Bud13p. *b,* complementation of Δ*bud13* strains with an empty vector, or plasmids expressing wild type or mutant Bud13p was monitored through the growth of serial dilutions of transformants on selective plates at the indicated temperatures. *c*, Western blot showing the level of wild type and mutant Bud13p is detected through the fused TAP tag. *d,* complementation of a Δ*snu17* strain was performed as for *bud13* in *b. e,* the levels of wild type and mutant Snu17p were monitored by detection of the TAP tag by Western blotting.

To characterize the Snu17p-Bud13p interaction *in vivo* we performed mutational analysis in *S. cerevisiae*. We first tested the impact of mutations on complex formation *in vitro* by co-expressing His_6_-Snu17p, Bud13p, and Pml1p encoded by an operon in *E. coli* and assessing complex assembly by purification on Ni-NTA (4). A double or triple mutant around the conserved Trp residue (R231D,W232A or R231D,W232A,D233N) of Bud13p abolished Bud13p incorporation in the RES complex (Fig. 2*a*). To assay the *in vivo* function, a yeast strain lacking the *bud13* gene (Δ*bud13*) was transformed with a vector carrying either the wild type Bud13p tagged with the TAP tag, or its double or triple mutant derivatives. Cells carrying the vector without insert were used as a negative control. At 30 °C, the growth of both mutants was only mildly affected (slightly smaller colony sizes than wild type cells, Fig. 2*b*). Consistent with previous observations deletion of *bud13* resulted in a strong phenotype at 37 °C (3). In this condition, both mutants were barely different from cells transformed with the vector indicating that they were not functional. Transformation of the same *bud13* mutants in a wild type strain did not result in slow growth demonstrating that they were not functional rather than acting as dominant negatives. Moreover, growth deficiency was not due to a decreased expression of the mutant proteins (detected by Western blot analyses of the TAP tag) (Fig. 2*c*) excluding that the mutations acted simply by destabilizing the proteins. These data indicate that Trp^232^ of Bud13p indeed is important for the interaction with Snu17p but that flanking residues also contribute to the binding.

Snu17p mutants were designed based on sequence homology with UHM domains that utilize a hydrophobic pocket between the two helices of the UHM domain to accommodate the invariant Trp of the ULM ligand. We targeted Snu17p residues corresponding to the Arg-*X*-Phe motif (^95^FKI^97^). A triple mutant (F95A,K96D,I97A) did not disrupt RES complex formation as assayed by co-expression of the proteins in *E. coli* followed by Ni-NTA purification (Fig. 2*a*). We also tested the ability of this mutant to rescue the growth phenotype of Δ*snu17* yeast cells. The mutant grew well at all temperatures tested (Fig. 2*d*) both in the mutant and wild type strain backgrounds. Western blotting confirmed that the mutant was well expressed (Fig. 2*e*). These results suggest that Snu17p may use a different interaction surface than canonical UHM proteins to bind the Bud13p ligand.

##### Biophysical Analysis of Snu17p-Bud13p Complex

**FIGURE 3.**
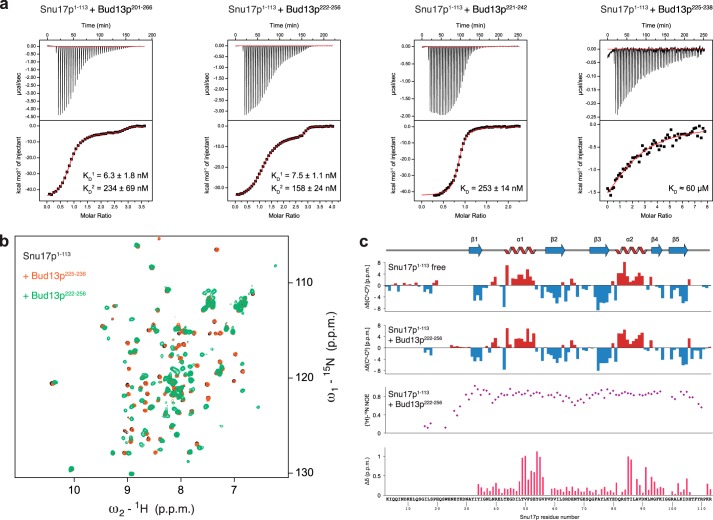
**ITC and NMR analysis of Snu17p binding to Bud13p.** *a,* four different Bud13p peptides containing the invariant Trp residue were tested by ITC titrations for Snu17p binding. Binding curves for a 66-mer, a 35-mer used in the accompanying structural studies, a 22-mer, and a 14-mer Bud13p peptide, are depicted from *left to right*. Dissociation constants obtained by fitting the isotherms after subtracting the heat of dilution are listed. *b,* overlay of ^1^H,^15^N HSQC spectra of free Snu17p (residues 1–113, *black*) and after titration with the shortest Bud13p ligand (residues 225–238, *orange*) or with the optimal Bud13p peptide (residues 222–256, *green*). *c,* secondary ^13^C chemical shifts Δδ(^13^C^α^)-Δδ(^13^C^β^) for Snu17p free and bound to Bud13p-(222–256), {^1^H}-^15^N heteronuclear NOE of the Snu17p-Bud13p complex, and chemical shift perturbations of Snu17p amide signals (recorded on a sample of ^15^N-labeled Snu17 residues 25–113) upon binding to Bud13p-(222–256) are plotted *versus* the amino acid sequence of Snu17p. Secondary structural elements of Snu17p are indicated on *top*.

To characterize the unexpected molecular features of the Snu17p-Bud13p interaction we proceeded with biophysical and structural characterization of the binary complex (Fig. 3). We first used a construct comprising the Snu17p RRM and an N-terminal unstructured (see below) extension (Snu17p, residues 1–113) as this domain was shown to be sufficient for the interaction with Bud13p (4, 6). We tested binding to a series of Bud13p fragments to identify the minimum binding region in Bud13p (Fig. 3, *a* and *b*). NMR titrations confirmed binding to a Bud13p peptide encompassing residues 201–266 as has been shown previously (20). Addition of this peptide to recombinant ^15^N-labeled Snu17p induced extensive chemical shift perturbations (CSPs) in ^1^H,^15^N correlation spectra but also led to precipitation of the sample. Similar CSPs were observed with a shorter Bud13p peptide (residues 222–256) but proteins remained in solution (Fig. 3*b*). An even shorter peptide (residues 221–242) affected the same Snu17p residues but to a lesser extent compared with the longer ones. Finally, a very short Bud13p peptide (residues 225–238) hardly induced any NMR spectral changes for Snu17p, even though this peptide has the typical length of ULM ligands and harbors the invariant Trp (Fig. 3*b*). Thus, the NMR data indicate that recognition of an ULM-like motif with its central Trp residue is not sufficient for Bud13p-Snu17p complex formation. This further demonstrates a different recognition mode involving additional residues present in the longer Bud13p peptides.

The Snu17p-Bud13p interaction was further characterized by ITC to quantitate the binding affinity and characterize the thermodynamic features of the interaction. The binding isotherms of the two longer peptides show low nanomolar binding affinity. The calorimetric analysis also suggests a second interaction at large excess of Bud13p, however, with a lower affinity with binding constants separated by 2 orders of magnitude (Fig. 3*a*), potentially reflecting an additional nonspecific interaction. In any case, the ITC data corroborate NMR findings that the additional residues present in the very long Bud13p peptide do not contribute to Snu17p binding when compared with the Bud13p-(222–256) peptide. The low nanomolar binding affinities are in very good agreement with the thermodynamic binding parameters of the full-length Snu17p-Bud13p complex (6). The ITC titration of Snu17p with the shorter Bud13p peptide (residues 221–242) could be fitted to a single binding site with a dissociation constant 50 times weaker compared with the longer ligands (Fig. 3*a*). The lower affinity is in accordance with quantitative information obtained from NMR chemical shift titration analysis for the Bud13p ligands (data not shown). For the shortest Bud13p peptide, the signal to noise ratio of the ITC signal was too small to make a precise determination of its binding constant, indicating that it binds very weakly consistent with the NMR titrations (Fig. 3, *a* and *b*). Based on NMR and ITC data we conclude that residues 222–256 of Bud13p encompass the minimum interacting region with Snu17p.

Next, we assigned the backbone chemical shifts of Snu17p (residues 1–113) when free and in a stoichiometric complex with unlabeled Bud13p (residues 222–256). Chemical shift analysis shows that Snu17p contains the expected secondary structure elements of an RRM domain in both free and bound forms (Fig. 3*c*), *i.e.* the secondary structure is preserved upon Bud13p binding. The first 25 residues of Snu17p do not exhibit any secondary structure and {^1^H}-^15^N heteronuclear NOE data show that the N terminus preceding the RRM domain is highly flexible (Fig. 3*c*). The residues showing large chemical shift changes, induced by Bud13p binding, map on the two helices plus adjacent loop regions of the Snu17p RRM-fold indicating a large interaction surface (Fig. 3*c*). These CSPs are distinct from what would be expected for a canonical UHM-ULM interaction, demonstrating that Snu17p and Bud13p use a different binding interface. Virtually identical NMR CSPs induced by Bud13p binding to a shorter Snu17p protein (residues 25–113) demonstrated that the flexible Snu17p N terminus does not participate in the interaction (data not shown).

**FIGURE 4.**
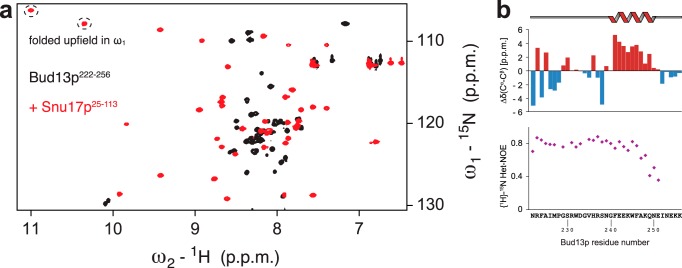
**NMR analysis of Bud13p ligand (residues 222–256) binding to Snu17p.** *a*, overlay of the ^1^H,^15^N HSQC spectra of the Bud13p ligand free and when bound to Snu17p. *b,* secondary ^13^C chemical shifts Δδ(^13^C^α^-^13^C^β^) and {^1^H}-^15^N heteronuclear NOE data for the Bud13p ligand when bound to Snu17p. The last 5 residues of Bud13p have negative NOE values and are not shown. The short helix in the C-terminal region of the peptide is indicated on *top*.

The Snu17p-Bud13p interaction was also characterized by monitoring NMR signals of ^15^N-labeled Bud13p (residues 222–256) in complex with unlabeled Snu17p. Binding to Snu17p induces extensive chemical shift changes in Bud13p spectra (Fig. 4*a*). Secondary chemical shift analysis indicates the presence of an α-helical segment at the C terminus of the bound Bud13p peptide (Fig. 4*b*). Steady state {^1^H}-^15^N heteronuclear NOE data show that a large portion of the peptide is rigid in the complex suggesting that the region comprising Bud13p residues 222–246 contributes to the Snu17p binding interface directly or become less flexible due to complex formation (Fig. 4*b*). Interestingly, the C-terminal helical region remains partially flexible.

##### Structure of the Snu17p-Bud13p Complex

**FIGURE 5.**
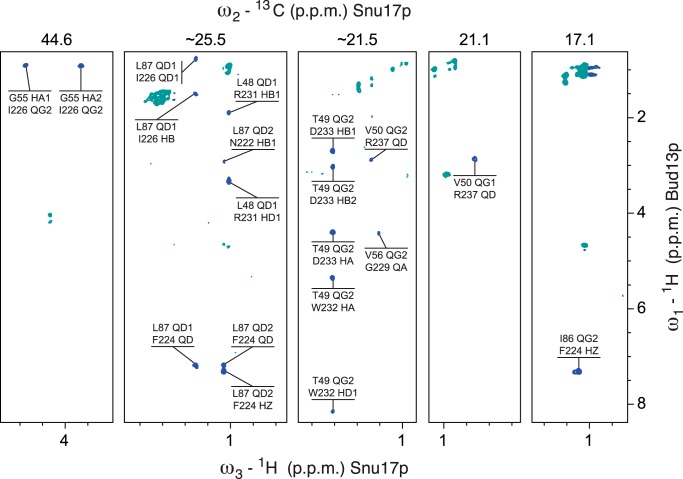
**^1^H (ω_3_),^1^H (ω_1_) planes from ^13^C/^15^N-filtered, ^13^C-edited NOESY-HSQC spectra in the binary complex composed of doubly labeled Snu17p and unlabeled Bud13p.** The ^13^C chemical shifts (ppm) of the selected ω_2_ planes are indicated on *top*. The experiment selects NOE cross-peaks between protons bound to ^12^C or ^14^N in Bud13p and protons bound to ^13^C in Snu17p, providing exclusively intermolecular restraints. For each cross-peak the upper and lower atom name refers to Snu17p and Bud13p assignments, respectively.

**FIGURE 6.**
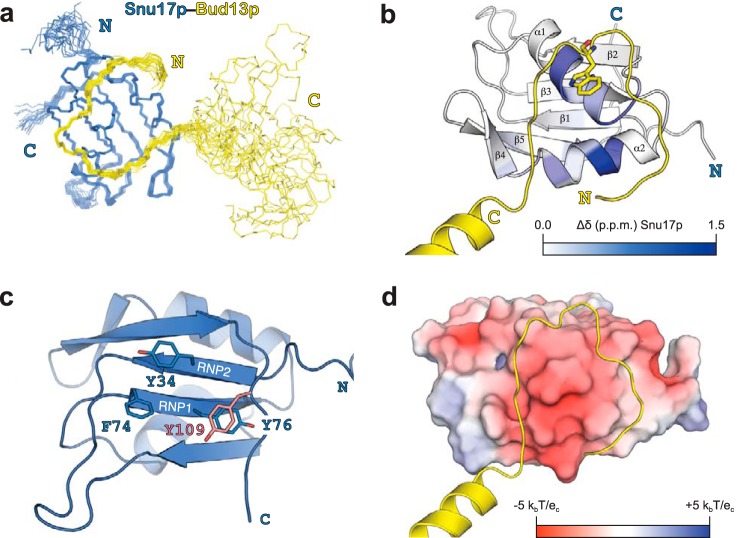
**Structure of the Snu17p-Bud13p complex.** *a*, overlay of the 20 lowest energy structures of Snu17p (*blue*) in complex with Bud13p (*yellow*). The N and C terminus of each protein is annotated. *b,* schematic representation of the Snu17p-Bud13p complex with amide CSP induced by Bud13p binding mapped onto the structure with a color gradient from *white* to *blue* with increasing CSP. The invariant Trp residue of Bud13p is shown as stick representation. *c*, the RNP signature motifs of Snu17p (Tyr^34^, Phe^74^, Tyr^76^) are conserved but not entirely exposed to the solvent. *d,* electrostatic surface of Snu17p with negative and positive charges colored *red* and *blue*, respectively.

We then determined the solution structure of the Snu17p-Bud13p complex (comprising Snu17p residues 25–113 and Bud13p residues 222–256) by heteronuclear triple-resonance NMR spectroscopy using subunit selectively isotope-labeled samples. The two proteins were prepared separately and co-purified by size exclusion chromatography after mixing an excess of Bud13p with Snu17p. In this way we were able to selectively label each of the proteins in the complex and determine 129 intermolecular distance restraints, derived from ^13^C- and ^15^N-edited NOESY HSQC spectra as well as ^13^C/^15^N-filtered and ^13^C-edited NOESY-HSQC spectra (Fig. 5). The structure of the complex is well defined and based on 2607 experimental distance restraints (Fig. 6). A summary of the structural and restraint statistics is given in Table 1. The ensemble of the 20 lowest energy structures obtained after water refinement is shown in Fig. 6*a*.

**TABLE 1:** **NMR and refinement statistics for the Snu17p-Bud13p complex**

NMR distance and dihedral restraints	
**Distance restraints**	
Total NOE	2607
Intra-residue	636
Inter-residue	1971
Sequential (|*i* – *j*| = 1)	682
Medium range (|*i* – *j*| < 4)	413
Long range (|*i* – *j*| > 5)	747
Intermolecular	129
Total dihedral angle restraints	177
φ	87
ψ	90

**Structure statistics**	
Violations (mean ± S.D.)	
Distance restraints (Å)	0.011 ± 0.00
Dihedral angle restraints (°)	0.767 ± 0.07
Maximum dihedral angle violation (°)	5.79
Maximum distance restraint violation (Å)	0.34
Deviations from idealized geometry	
Bond lengths (Å)	0.011 ± 0.00
Bond angles (°)	1.206 ± 0.03
Impropers (°)	1.297 ± 0.06
Average pairwise root mean square deviation*^a^* (Å)	
Heavy	1.15 ± 0.17
Backbone	0.55 ± 0.12
Ramachandran plot statistics (%)	
Residues in most favored regions	93.4
Residues in additionally allowed regions	6.6

*^a^* Pairwise root mean square deviation was calculated among 20 refined structures for residues 30–109 of Snu17p and 222–239 of Bud13p.

In the complex Snu17p adopts an RRM-fold with two α-helices packing on one surface of an antiparallel β-sheet (Fig. 6*b*). The Snu17p structure superimposes very well with UHM family members (root mean square deviations 1.5–1.8 Å for 72 C_α_ atoms against the proteins listed in Fig. 1*a*). Noteworthy, although most UHM family members lack the conserved aromatic residues in RNP1 and RNP2 that mediate RNA binding in canonical RRMs, Snu17p has retained both RNPs. However, the structure shows that Tyr^76^ (RNP1) is not solvent exposed but rather stacks parallel with another tyrosine (Tyr^109^) located at the C-terminal extension of the RRM-fold (Fig. 6*c*). The partial solvent protection of the RNP1 motif may imply potential regulation of RNA binding by Snu17p in the RES complex.

Bud13p binds to the helical surface of the RRM domain of Snu17p, consistent with chemical shift mapping data (Fig. 6*b*). Although Bud13p contains the invariant Trp residue of ULMs, the interaction is distinct from the canonical UHM-ULM recognition. Bud13p assumes an extended conformation and forms an arch around the Snu17p RRM above the two Snu17p helices. Two glycine residues flanking the invariant Trp that are not present in other ULMs change the backbone direction of the Bud13p on the surface of Snu17p. Eighteen amino acids of Bud13p interact with Snu17p forming a U-turn that crosses the helical interface of Snu17p (Fig. 6*b*). The invariant Trp is located in the middle of the turn and is recognized together with the flanking interacting residues of Bud13p on the surface of Snu17p (Fig. 6*b*). The C-terminal helix of the Bud13p ligand does not contribute to the binding interface and its orientation is not defined by experimental data. This suggests that it is flexible in solution, consistent with reduced heteronuclear {^1^H}-^15^N NOE values (Fig. 4*b*). The Snu17p-Bud13p binding surface area covers ∼800 Å^2^, and is thus significantly larger compared with any UHM-ULM interaction described (Fig. 6*d*).

**FIGURE 7.**
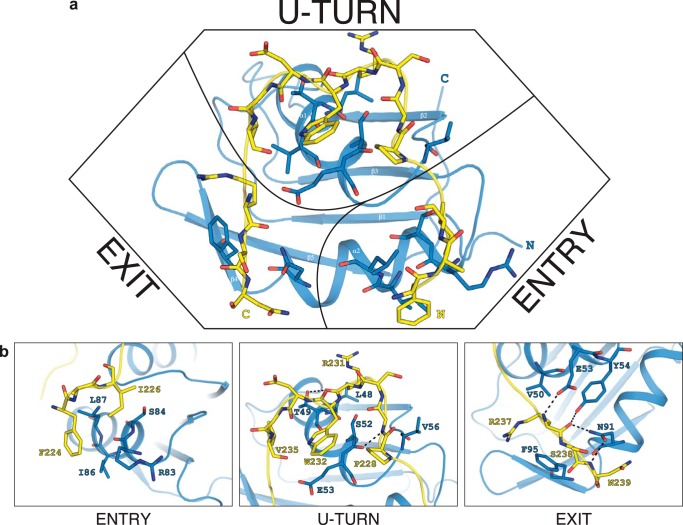
**Details of the intermolecular contacts in the Snu17p-Bud13p complex.** *a,* the Bud13p peptide forms an arch around the helical binding surface of Snu17p. *b,* close-up views of the interaction at the entry point (*left*), the U-turn (*middle*), and the exit point (*right*) of the Bud13p peptide shown in *a*. Interacting residues are annotated and colored *blue* for Snu17p and *yellow* for Bud13p. Intermolecular hydrogen bonds to the Bud13p backbone are indicated by *dashed lines*.

The binding interface between Snu17p and Bud13p is mainly stabilized by hydrophobic interactions. The extensive bipartite hydrophobic interface includes the whole stretch of residues from Asn^222^ to Asn^239^ of Bud13p and the dorsal helical face of the Snu17p RRM domain. The apolar contacts are supported by electrostatic interactions involving mainly backbone amide groups of Bud13p (Fig. 6*d*). The entire backbone of the Bud13p stretch is involved in intermolecular contacts but certain amino acids engage their side chains as well, thus contributing more to the interaction. Three regions of higher significance can be discerned based on intermolecular NOE data and the relative contribution to the total interaction surface (Fig. 7*a*). The first spot is at the N terminus of Bud13p where it enters the interaction surface and passes above helix α2 of Snu17p. Phe^224^ and Ile^226^ of Bud13p exhibit mainly hydrophobic contacts with the helical interface lined by Snu17p Arg^83^, Ser^84^, Ile^86^, and Leu^87^ (Fig. 7*b*). The second binding hot spot is at the U-turn of Bud13p. Bud13p residues from Pro^228^ to Val^235^ encircle the indole ring of the invariant Bud13p Trp^232^. The curved conformation of the Bud13p peptide is supported by many intramolecular NOEs between Bud13p Trp^232^ and Pro^228^ on one side and Val^235^ on the other side. The presence of Gly^229^ following Pro^228^ and Gly^234^ preceding Val^235^ provide the conformational flexibility required for reversing the backbone course of Bud13p. In the U-turn configuration Pro^228^, Arg^231^, Trp^232^, and Val^235^ of Bud13p form a continuous surface that interacts with Snu17p helix α1 (residues Leu^48^, Thr^49^, Ser^52^, and Glu^53^), as well as Snu17p Val^56^ in the linker following helix α1 (Fig. 7*b*). The third interface involves Snu17p Val^50^, Phe^95^, and Asn^91^, which contact Bud13p Arg^237^, Ser^238^, and Asn^239^, respectively, at the crevice between the two α-helices of the Snu17p RRM (Fig. 7*b*). This interface is further stabilized by hydrogen bonds between the Bud13p backbone and Snu17p side chains. At this point Bud13p ligand exits the binding interface with Snu17p and assumes a helical conformation.

## DISCUSSION

The solution structure of Snu17p-Bud13p reveals a novel binding mode that has not been observed in other RRM-UHM peptide complexes. The structural findings are consistent with our initial mutagenesis analysis, which was guided by the recognition features described for the UHM family. We found that a double or triple mutant around the invariant Trp residue of Bud13p (R231D,W232A or R231D,W232A,D233N) disrupted Snu17p binding *in vitro*, and impacted on normal cell growth of transformants, whereas the single W232A substitution did not prevent complex formation (4), in contrast to canonical UHM-ULM interactions (18, 30). On the other hand, a triple mutant of Snu17p residues that correspond to the UHM Arg-*X*-Phe motif (F95A,K96D,I97A) had no effect in formation of the RES complex and restored a growth phenotype similar to wild type. These findings indicated an unexpected architecture of recognition that is distinct from the established UHM-ULM mode.

**FIGURE 8.**
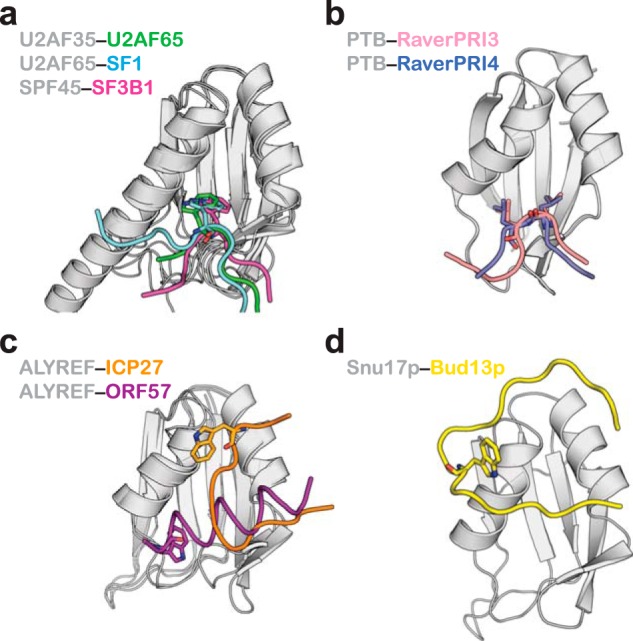
**Comparison of ligand binding modes of the UHM/RRM-fold.** In all panels the UHM/RRM domains are shown in the same orientation. *a,* three examples of the canonical UHM-ULM interaction. *b*, PTB binding to Raver1 ligands, which lack a Trp residue. *c,* two different binding modes of peptide partners with a Trp residue by ALYREF protein. *d,* the novel Bud13p peptide recognition by Snu17p.

Several structures have revealed the main features that determine UHM-ULM interactions (16–19, 30, 31). UHM domains typically lack one or more aromatic residues in their RNP sequences, they have an acidic helix α1, and contain an Arg-*X*-Phe motif following the second helix α2. The Arg-*X*-Phe motif recognizes ULM sequences that contain an invariant Trp residue that is usually preceded by basic residues. The critical Trp is inserted into a hydrophobic cleft formed between the two helices of the UHM-fold with the ULM peptide oriented perpendicular to the two helices (Fig. 8*a*). In general, UHM domains do not interact with RNA as often the RNA-binding β-sheet surface on the opposite side of the RRM-fold is occluded by a C-terminal helix.

An interesting variation of the UHM-ULM theme is the interaction between the PTB protein and its corepressor Raver1 (32). The consensus signature motif of Raver1 ligands do not harbor the Trp residue but instead a pair of Leu residues. The PTB structures in complex with two peptide motifs from Raver1 (33) show that the Leu residues substitute for the Trp recognition seen in UHM-ULM binding and interact with a shallow groove formed at the same location on PTB RRM2 domain (Fig. 8*b*). Interestingly, it has been reported that the PTB RRM2 domain can bind simultaneously Raver1 and RNA, a distinctive feature that has not been described for other UHM domains.

The most notable exception of RRM binding to Trp containing ligands has been reported for the cellular mRNA export factor ALYREF (34). Two structures have been determined of the ALYREF RRM domain in complex with viral peptides, both different from the UHM-ULM mode (Fig. 8*c*). The ICP27 peptide binds ALYREF RRM in an extended conformation along the groove formed by the helices (34). The Trp residue is located at the upper edge of the first ALYREF helix and contributes to the total interaction surface. The ORF57 peptide on the other hand adopts a helical conformation running almost perpendicular to the helices of ALYREF (35). The Trp residue is positioned at one end of the helix and makes hydrophobic contacts with ALYREF along with other residues of the helical peptide. Mutational analysis indicates that in ORF57 the Trp residue is important for ALYREF recognition, whereas in ICP27 it is not as crucial as has been demonstrated for true UHM-ULM pairs.

The structure of Snu17p-Bud13p complex presented here displays yet another distinct binding topology that has not been described before, further adding to the diversity of RRM-ligand interactions. A large portion of the Bud13p ligand is engaged in contacts around the helical surface of Snu17p. The Trp residue is located in the middle of a U-turn configuration that collectively contacts the first helix of Snu17p (Fig. 8*d*). Preceding and following residues to the U-turn are perpendicular to the second helix of Snu17p and contribute to binding. The importance of these residues to the interaction, which are rather distant to the invariant Trp, has been confirmed by *in vitro* binding studies designed to identify the minimal interacting region on Bud13p. Notably, key residues in Bud13p involved in Snu17p binding are conserved (data not shown) in other orthologs including the human homolog of Bud13p, suggesting that the novel mode of RRM-peptide interaction is conserved.

The yeast RES complex facilitates the splicing of some introns and participates in the retention of unspliced pre-mRNAs in the nucleus. The absence of a functional RES complex in yeast results in slow growth, particularly at high temperature, due to reduced splicing (3). Our data show that preventing the association of Bud13p with Snu17p impairs cell growth without affecting protein expression levels. This demonstrates that individual proteins when simultaneously present are unable to perform the RES activity and thus that assembly of the RES complex is essential for function. It is conceivable that to perform its task the RES complex recognizes specific RNA sequences through its Snu17p subunit. The Snu17p-Bud13p structure shows that Bud13p binding does not interfere with the RNP sequences of Snu17p. Snu17p could thus be involved in RNA recognition as shown for canonical RRM domains (13, 14). However, in Snu17p the first RNP motif is partially occluded from the solvent by the C-terminal tail of the RRM-fold. We envisage that Pml1p may regulate RNA binding of Snu17p in the functional ternary RES complex. The binding of Pml1p, which requires the Snu17p C-terminal portion in addition to the RRM-fold, could induce structural rearrangements that might completely expose the RNP motifs of Snu17p. Future studies with the ternary RES complex and RNA should shed light to this hypothesis and increase our understanding for RES function in RNA splicing and nuclear retention of pre-messenger transcripts.

## Footnotes

6 The abbreviations used are:

snRNP: small nuclear ribonucleoprotein particle
RRM: RNA recognition motif
ITC: isothermal titration calorimetry
CSP: chemical shift perturbation
Ni-NTA: nickel-nitrilotriacetic acid
HSQC: heteronuclear single quantum coherence.
